# Identification of afatinib-associated ADH1B and potential small-molecule drugs targeting ADH1B for hepatocellular carcinoma

**DOI:** 10.3389/fphar.2023.1166454

**Published:** 2023-05-09

**Authors:** Yongxu Zhou, Liang Yu, Peng Huang, Xudong Zhao, Risheng He, Yunfu Cui, Bo Pan, Chang Liu

**Affiliations:** ^1^ Department of General Surgery, The Fourth Affiliated Hospital of Harbin Medical University, Harbin, China; ^2^ The Key Laboratory of Myocardial Ischemia, Ministry of Education, Harbin Medical University, Harbin, China; ^3^ Department of Hepatopancreatobiliary Surgery, Second Affiliated Hospital of Harbin Medical University, Harbin, China; ^4^ Department of Pathology, School of Clinical Medicine, Li Ka Shing Faculty of Medicine, The University of Hong Kong, Hong Kong, China; ^5^ Department of Hepatobiliary Surgery, Mudanjiang Tumor Hospital, Mudanjiang, China; ^6^ Department of Medical Oncology, Harbin Medical University Cancer Hospital, Harbin, China

**Keywords:** hepatocellular carcinoma, afatinib, ADH1B, CellMiner, small-molecule drugs, methylation

## Abstract

**Background:** Afatinib is an irreversible epidermal growth factor receptor tyrosine kinase inhibitor, and it plays a role in hepatocellular carcinoma (LIHC). This study aimed to screen a key gene associated with afatinib and identify its potential candidate drugs.

**Methods:** We screened afatinib-associated differential expressed genes based on transcriptomic data of LIHC patients from The Cancer Genome Atlas, Gene Expression Omnibus, and the Hepatocellular Carcinoma Database (HCCDB). By using the Genomics of Drug Sensitivity in Cancer 2 database, we determined candidate genes using analysis of the correlation between differential genes and half-maximal inhibitory concentration. Survival analysis of candidate genes was performed in the TCGA dataset and validated in HCCDB18 and GSE14520 datasets. Immune characteristic analysis identified a key gene, and we found potential candidate drugs using CellMiner. We also evaluated the correlation between the expression of ADH1B and its methylation level. Furthermore, Western blot analysis was performed to validate the expression of ADH1B in normal hepatocytes LO2 and LIHC cell line HepG2.

**Results:** We screened eight potential candidate genes (ASPM, CDK4, PTMA, TAT, ADH1B, ANXA10, OGDHL, and PON1) associated with afatinib. Patients with higher ASPM, CDK4, PTMA, and TAT exhibited poor prognosis, while those with lower ADH1B, ANXA10, OGDHL, and PON1 had unfavorable prognosis. Next, ADH1B was identified as a key gene negatively correlated with the immune score. The expression of ADH1B was distinctly downregulated in tumor tissues of pan-cancer. The expression of ADH1B was negatively correlated with ADH1B methylation. Small-molecule drugs panobinostat, oxaliplatin, ixabepilone, and seliciclib were significantly associated with ADH1B. The protein level of ADH1B was significantly downregulated in HepG2 cells compared with LO2 cells.

**Conclusion:** Our study provides ADH1B as a key afatinib-related gene, which is associated with the immune microenvironment and can be used to predict the prognosis of LIHC. It is also a potential target of candidate drugs, sharing a promising approach to the development of novel drugs for the treatment of LIHC.

## Introduction

Globally, liver cancer significantly increases the world’s cancer burden. Liver cancer ranks the sixth for incidence and the third for cancer-related death according to the Global Cancer Statistics 2020 (Sung et al., 2021). The most common histologic type of liver cancer is hepatocellular carcinoma (LIHC), and it is estimated that LIHC accounts for 75%–85% of all liver cancer cases in the world (Sung et al., 2021). Although non-viral risk factors including alcohol, metabolic syndrome, obesity, diabetes, and non-alcoholic fatty liver disease have had major impacts on the development of LIHC, hepatitis B virus (HBV) and hepatitis C virus (HCV) are still predominant viral causes of LIHC (McGlynn et al., 2021). Currently, first-line drugs such as sorafenib, lenvatinib, and nivolumab as well as second-line drugs including regorafenib and cabozantinib are commonly used systemic treatments for LIHC (Chen et al., 2020; Foerster and Galle, 2021). Unfortunately, most of the patients were insensitive to systemic treatments, and the 5-year overall survival of LIHC patients is approximately 19.6% and can even decline to 2.5% for advanced and metastatic patients (Chidambaranathan-Reghupaty et al., 2021). Therefore, it is essential to develop potential targets and effective candidate drugs for systemic treatments with the help of high-throughput sequencing technology at the molecular level to improve prognosis for patients with LIHC.

The epidermal growth factor receptor (EGFR), a transmembrane receptor tyrosine kinase, plays an important role in proliferation, differentiation, and survival and is involved in tumorigenesis, especially in lung cancer, breast cancer, and glioblastoma (Sun et al., 2018). Additionally, the tumor-promoting function of the activated EGFR in LIHC has been previously documented (Komposch and Sibilia, 2015). Meanwhile, the EGFR contributes to drug resistance in tumors (Jin et al., 2021). Afatinib is an FDA-approved irreversible blocker of the tyrosine kinase of the EGFR for treating advanced or metastatic non-small-cell lung cancer (NSCLC) (Wecker and Waller, 2018). It has been reported that afatinib inhibits epithelial–mesenchymal transition and tumorigenesis of LIHC cells via inactivation of extracellular signal-regulated kinase (ERK)-vascular endothelial growth factor (VEGF)/matrix metalloproteinase (MMP) 9 signaling (Chen et al., 2019). The combination of ethoxy-erianin phosphate and afatinib exerts synergistic effects on LIHC tumor growth and angiogenesis through VEGF/EGFR signaling (Chen et al., 2022). Moreover, a recent study demonstrated that the application of EGFR inhibitor WZ3146 and afatinib showed strong synergistic effects with cabozantinib on LIHC cells (Ma et al., 2022). These compelling pieces of evidence suggested that afatinib may have great potential as a treatment for LIHC. Thus, screening afatinib-associated genes may facilitate to mine potential targets and candidate drugs for LIHC patients.

In this study, we screened potential candidate genes of afatinib based on transcriptomic data of LIHC patients from The Cancer Genome Atlas (TCGA), GSE14520, the Hepatocellular Carcinoma Database (HCCDB), and the Genomics of Drug Sensitivity in Cancer (GDSC) 2 database. Through immune characteristics analysis, ADH1B was initially considered a key gene. Potential regulatory pathway analysis revealed the underlying mechanism of ADH1B in LIHC. We also found four potential small-molecule drugs significantly associated with ADH1B. Our study reveals ADH1B as a potential target for afatinib treatment and provides promising drugs targeting ADH1B.

## Materials and methods

### Data collection and pre-processing

We downloaded transcriptomic data and corresponding clinical information of LIHC patients from The Cancer Genome Atlas (TCGA) database (https://portal.gdc.cancer.gov/). The samples without survival time or survival status were eliminated from this study, keeping samples with survival time longer than 0 days. A total of 365 LIHC tissue samples and 50 para-carcinoma tissue samples were included.

We also downloaded GSE14520 from the Gene Expression Omnibus (GEO; https://www.ncbi.nlm.nih.gov/geo/) database. We converted the probe to a gene symbol. We removed normal tissues and eliminated samples without follow-up information or OS information, ensuring that all the samples had survival time greater than 0 days. A total of 242 LIHC tissue samples were collected.

From the Hepatocellular Carcinoma Database (HCCDB) (http://lifeome.net/database/hccdb/) (Lian et al., 2018), we acquired transcriptomic data and survival information of 212 LIHC tissue samples after removing normal tissues and those without follow-up information. Moreover, from the Genomics of Drug Sensitivity in Cancer 2 (GDSC2) database (https://www.cancerrxgene.org/) (Yang et al., 2012), we obtained afatinib treatment-related LIHC cell line expression profile data and half-maximal inhibitory concentration (IC50) information.

### Screening of potential candidate genes of afatinib

Datasets from TCGA were used for identifying differentially expressed genes (DEGs). We used the “limma” package (Ritchie et al., 2015) to screen the differential genes between tumor and para-carcinoma tissue samples under the threshold of |log2(fold change)| > 1 and false discovery rate (FDR) < 0.05. Next, univariate Cox regression analysis was performed to screen genes using the “survival” package (Therneau and Lumley, 2015) in TCGA, HCCDB18, and GSE14520 datasets. Furthermore, the correlation between these genes and the IC50 value of afatinib was analyzed to screen candidate genes. We performed Kyoto Encyclopedia of Genes and Genomes (KEGG) and Gene Ontology (GO) functional enrichment analyses using the “clusterProfiler” R package (Yu et al., 2012) for the common DEGs. The top 10 enriched pathways were selected if the FDR <0.05.

### Relationship of candidate genes with clinicopathological features and survival

Furthermore, we assessed the distribution of candidate genes in different clinicopathological features (T stage, stage, and grade) in the TCGA dataset. Differences were determined using the wilcox.test. The “survminer” package was used to determine the cutoff values of gene expression (). LIHC patients in the TCGA dataset were classified into the high-risk group and low-risk group, and Kaplan–Meier curves were generated for the two groups. The log-rank test was used to assess the significance of differences**.** Validation was performed in HCCDB18 and GSE14520 datasets.

### Identification of the key gene based on immune abnormalities

The immune score was predicted using the ESTIMATE algorithm (Yoshihara et al., 2013) in the TCGA dataset, and Spearmen correlation analysis was conducted to evaluate the correlation between candidate genes and immune score. From previous research (Charoentong et al., 2017), we collected 28 immune cells, the scores of which were calculated using the CIBERSORT algorithm (Chen et al., 2018). Spearman correlation analysis was utilized to assess the relationship between immune cells and ADH1B.

### Potential regulatory pathways of ADH1B

To study the potential function of ADH1B in the body, we used the “GSVA” package (Hänzelmann et al., 2013) to calculate the enrichment score of KEGG pathways. Significant pathways were selected based on the t.test. Next, enrichment analysis in the gene set of the HALLMARK database was performed using Gene Set Enrichment Analysis (GSEA). We collected 31 cell cycle progression (CCP)-related genes (Cuzick et al., 2011) and scored them using single-sample GSEA (ssGSEA). Additionally, we scored G1/S cell cycle, G2M checkpoint, and inflammation pathways from KEGG. Spearman correlation analysis was used to assess the relationship between ADH1B and pathways.

### Performance of ADH1B in pan-cancer

To evaluate the expression of ADH1B in pan-cancer, we downloaded the gene expression of pan-cancer from TCGA and GTEx using SangerBox (http://vip.sangerbox.com) (Shen et al., 2022). The differences were compared using the wilcox.test between tumor samples and normal samples. Based on a previous study (Liu et al., 2018), we obtained the survival time and survival status of pan-cancer and analyzed the relationship between ADH1B and survival in each cancer type using the “survival” package.

### Drug sensitivity analysis of ADH1B

Using CellMiner (https://discover.nci.nih.gov/cellminer/home.do), we screened the potential anticancer drugs in LIHC cells. Spearman analysis was performed to study the relationship between ADH1B and the sensitivity to small-molecule drugs. Under the *p*-value <0.05, potential drugs were selected to have significant correlation with ADH1B expression.

### Correlation between ADH1B expression and its methylation

We downloaded methylated data (450K) from TCGA and filled in missing values through the KNN algorithm. After extracting the peak value of the ADH1B gene, all peaks for each sample were averaged. We analyzed the correlation between ADH1B gene expression and the methylation value of the ADH1B gene using Pearson correlation analysis.

### Western blot analysis

LO2 and HepG2 were removed from the T25 culture flasks after 48 h of incubation. Proteins were separated by SDS-PAGE, and the separated proteins were subsequently transferred to PVDF (0.45) membranes. The membranes were first incubated with primary antibodies: ADH1B (17165-1-AP, Proteintech) and GADPH (60004-1-Ig, Proteintech) for one full day after blocking with 5% bovine serum albumin for 2 hours. After treating the membranes with secondary antibodies for 1 hour at room temperature, the protein bands were detected the next day with ECL solution (Billerica Millipore, United States). Protein band signals were sought using the ChemiDoc detection system (Bio-Rad, United States) and quantified using ImageJ (National Institutes of Health, United States). A *t*-test was used to compare the differences between LO2 and HepG2 cells. *p* < 0.05 was considered statistically significant.

## Results

### Screening of potential candidate genes of afatinib

**FIGURE 1:**
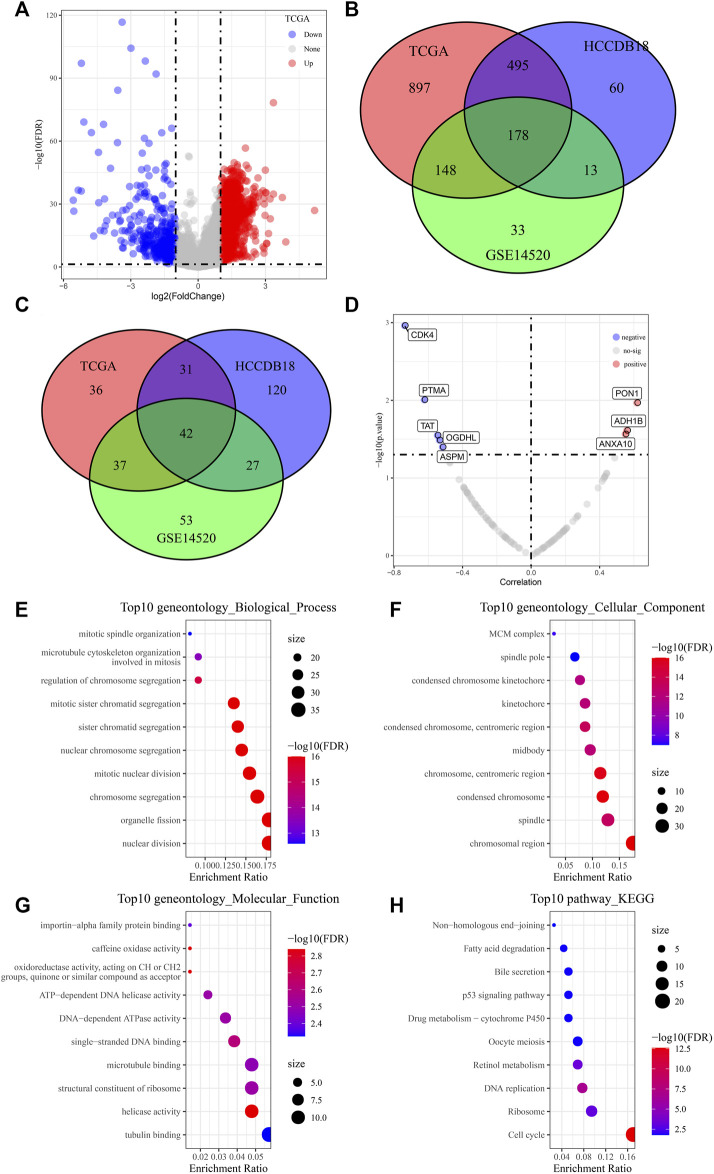
Screening of potential candidate genes of afatinib. **(A)** Volcano plots displaying 2356 differential upregulated genes and 462 downregulated differential genes between LIHC tumor and para-carcinoma tissue samples. **(B, C)** Venn diagram showing 178 risk genes and 42 protect genes among TCGA, HCCDB18, and GSE14520 datasets. **(D)** Scatter plots of correlation analysis between 220 genes and IC50 value of afatinib from LIHC cells in the GDSC2 database. **(E-H)** The GO and KEGG analysis of differentially expressed genes.

Through differential expression analysis, we screened 2818 differential genes, including 2356 upregulated genes and 462 downregulated genes, between tumor and para-carcinoma tissue samples (Figure 1A). Next, the Venn diagram showed 178 risk genes and 42 protect genes among TCGA, HCCDB18, and GSE14520 datasets (Figures 1B, C). Furthermore, we assessed the relationship between 220 genes and the IC50 value of afatinib. As displayed in Figure 1D, five candidate genes (CDK4, PTMA, TAT, OGDHL, and ASPM) were negatively correlated with the IC50 value of afatinib, while three candidate genes (PON1, ADH1B, and ANXA10) were positively correlated with the IC50 value of afatinib. Moreover, we performed functional enrichment analysis for common DEGs, and we found that these common DEGs were mainly enriched in mitotic spindle organization, nuclear division, fatty acid degradation, bile secretion, DNA replication, and cell cycle, indicating these DEGs might contribute to tumorigenesis by regulating these processes (Figures 1E–H).

### Distribution of candidate genes in clinicopathological features

**FIGURE 2:**
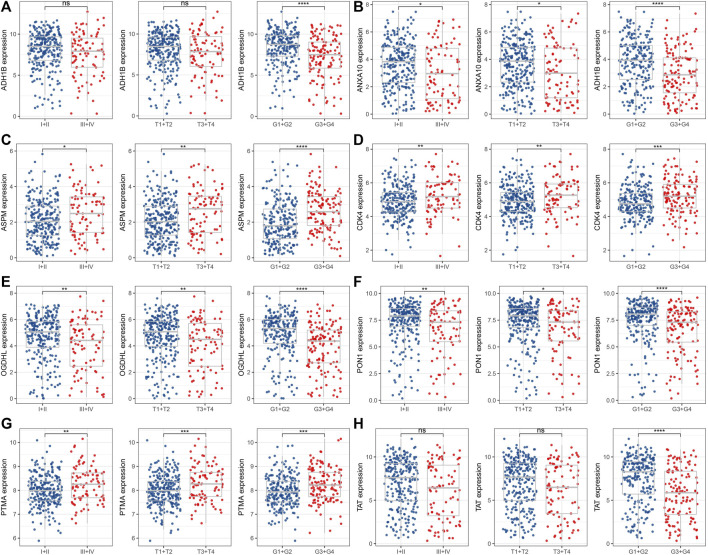
Distribution of eight candidate genes in clinicopathological features. The expression levels of ADH1B **(A)**, ANXA10 **(B)**, ASPM **(C)**, CDK4 **(D)**, OGDHL **(E)**, PON1 **(F)**, PTMA **(G)**, and TAT **(H)** between early and late stage or high and low grade. ns represents *p* > 0.05; *p < 0.05, **p < 0.01, ***p < 0.001, and ****p < 0.0001.

We assessed the distribution of candidate genes in different clinicopathological features (T stage, stage, and grade) in the TCGA dataset. We found that the expression of ADH1B was significantly decreased in patients with G3+G4 (Figure 2A); ANXA10, OGDHL, PON1, and TAT were highly expressed in patients with early stage and low grade (Figures 2B, E, F, H), whereas the expression of ASPM, CDK4, and PTMA was remarkably unregulated in patients with late stage and high grade (Figures 2C, D, G).

### Survival analysis of candidate genes

**FIGURE 3:**
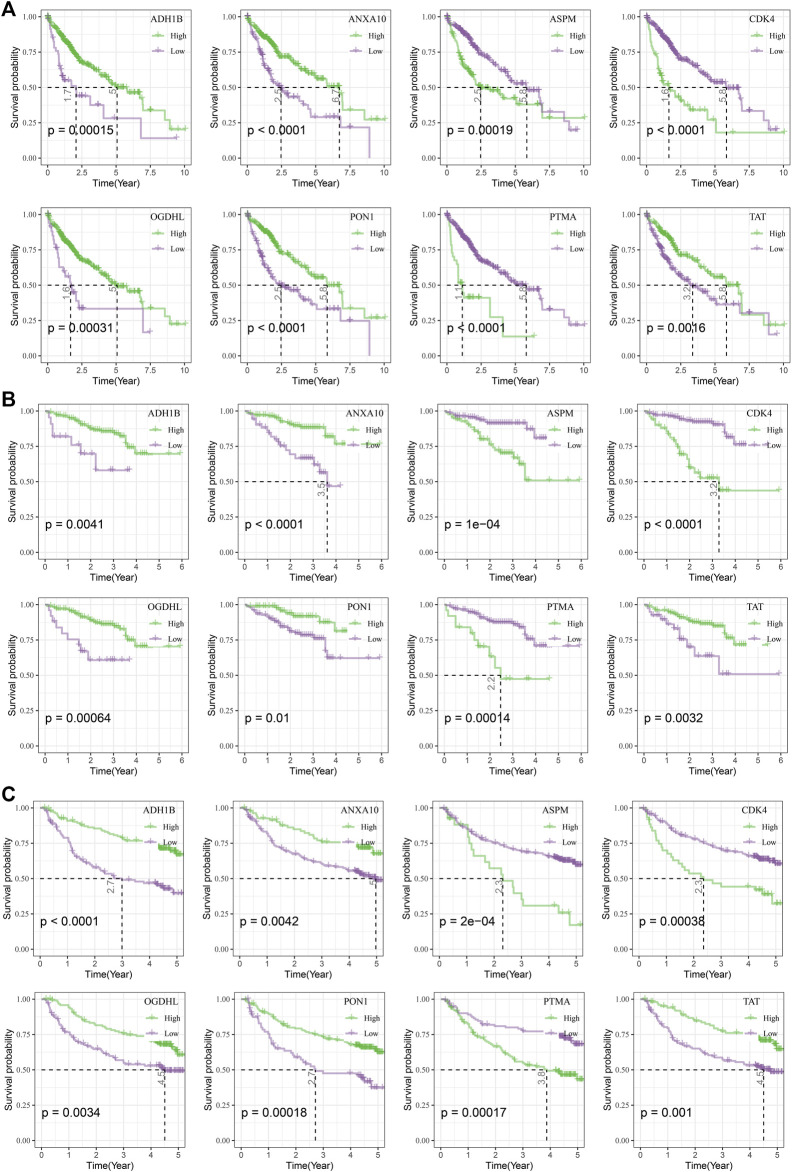
Survival analysis of candidate genes. **(A)** Kaplan–Meier curves of eight candidate genes in the TCGA dataset. **(B)**, Kaplan–Meier curves of eight candidate genes in the HCCDB18 dataset. **(C)** Kaplan–Meier curves of eight candidate genes in the GSE14520 dataset.

To analyze the relationship between candidate genes and survival, we performed survival analysis of eight candidate genes in TCGA, HCCDB18, and GSE14520 datasets. Patients in the TCGA dataset with a higher expression of ASPM (*p* = 0.00019), CDK4 (*p* < 0.0001), and PTMA (*p* < 0.0001) exhibited poorer prognosis compared with those with lower expression of ASPM, CDK4, and PTMA, while those with lower ADH1B (*p* = 0.00015), ANXA10 (*p* < 0.0001), OGDHL (*p* = 0.00031), PON1 (*p* < 0.0001), and TAT (*p* = 0.0016) had unfavorable prognosis (Figure 3A). Similar results could be found in HCCDB18 and GSE14520 datasets (Figures 3B, C).

### Identification of key genes based on immune abnormalities

**FIGURE 4:**
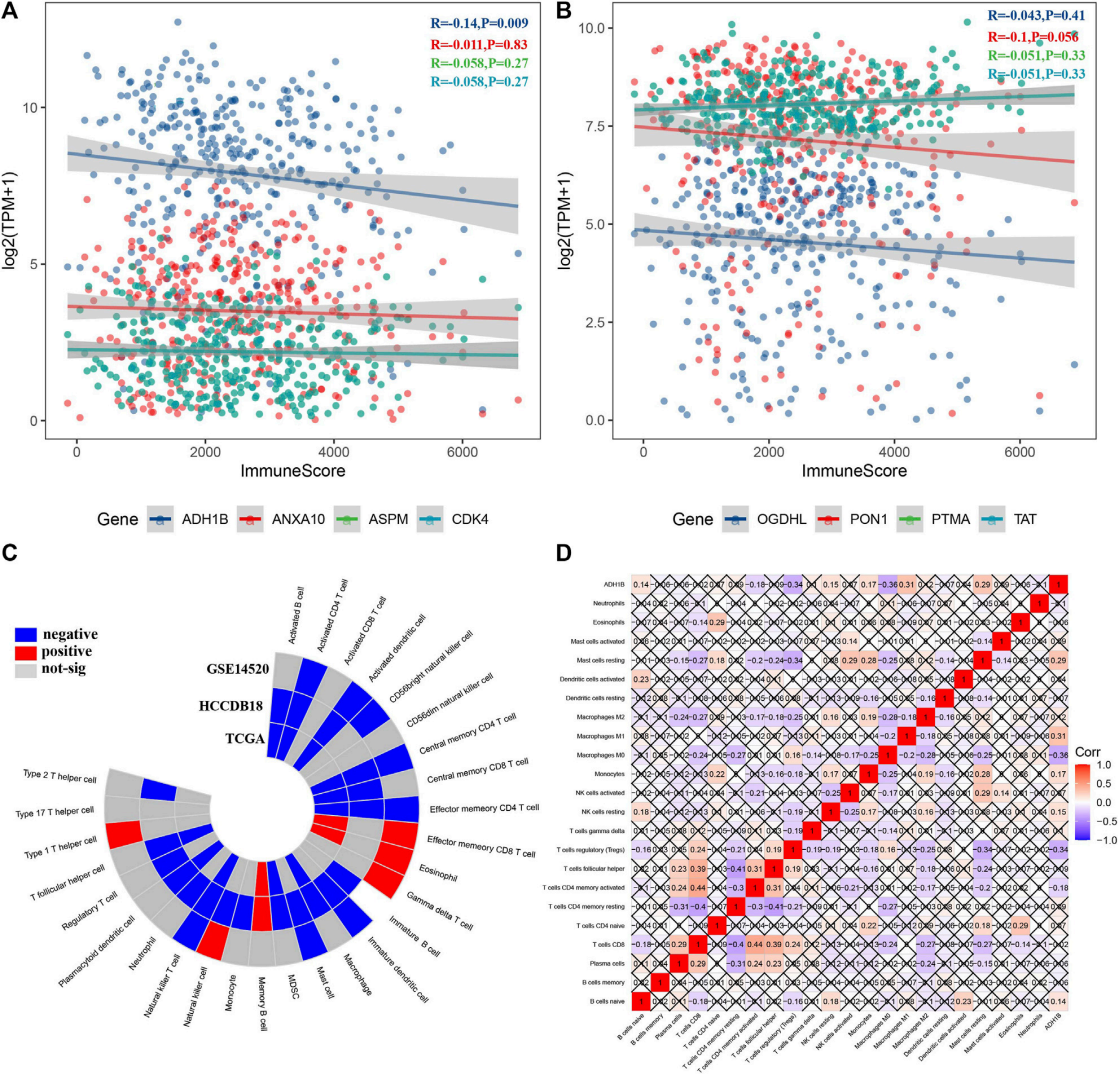
Identification of key genes based on immune abnormalities. **(A, B)** Among eight candidate genes, ADH1B was negatively correlated with the immune score. **(C)** ADH1B was significantly correlated with some immune cells among 28 immune cells. **(D)** Correlation heatmap displayed ADH1B correlated with several immune cells among 22 immune cells.

Furthermore, we analyzed the relationship between candidate genes and immune score and found that ADH1B was significantly negatively correlated with the immune score (R = −0.14, *p* = 0.009) (Figures 4A, B). Additionally, ADH1B was positively correlated with effector memory CD8 T cells, eosinophils, gamma delta T cells, memory B cells, and type 1 T helper cells in GSE14520, HCCDB18, or TCGA, while ADH1B was negatively correlated with several immune cells such as activated CD4 T cells, activated dendritic cells, central memory CD4 T cells, effector memory CD4 T cells, macrophage, mast cells, and MDSC (Figure 4C). Moreover, ADH1B had a positive correlation with resting mast cells, macrophage M1, monocytes, and resting NK cells; ADH1B was negatively correlated with macrophage M0, regulatory T cells (Tregs), and CD4 memory activated T cells (Figure 4D).

### Potential regulatory pathways of ADH1B

**FIGURE 5:**
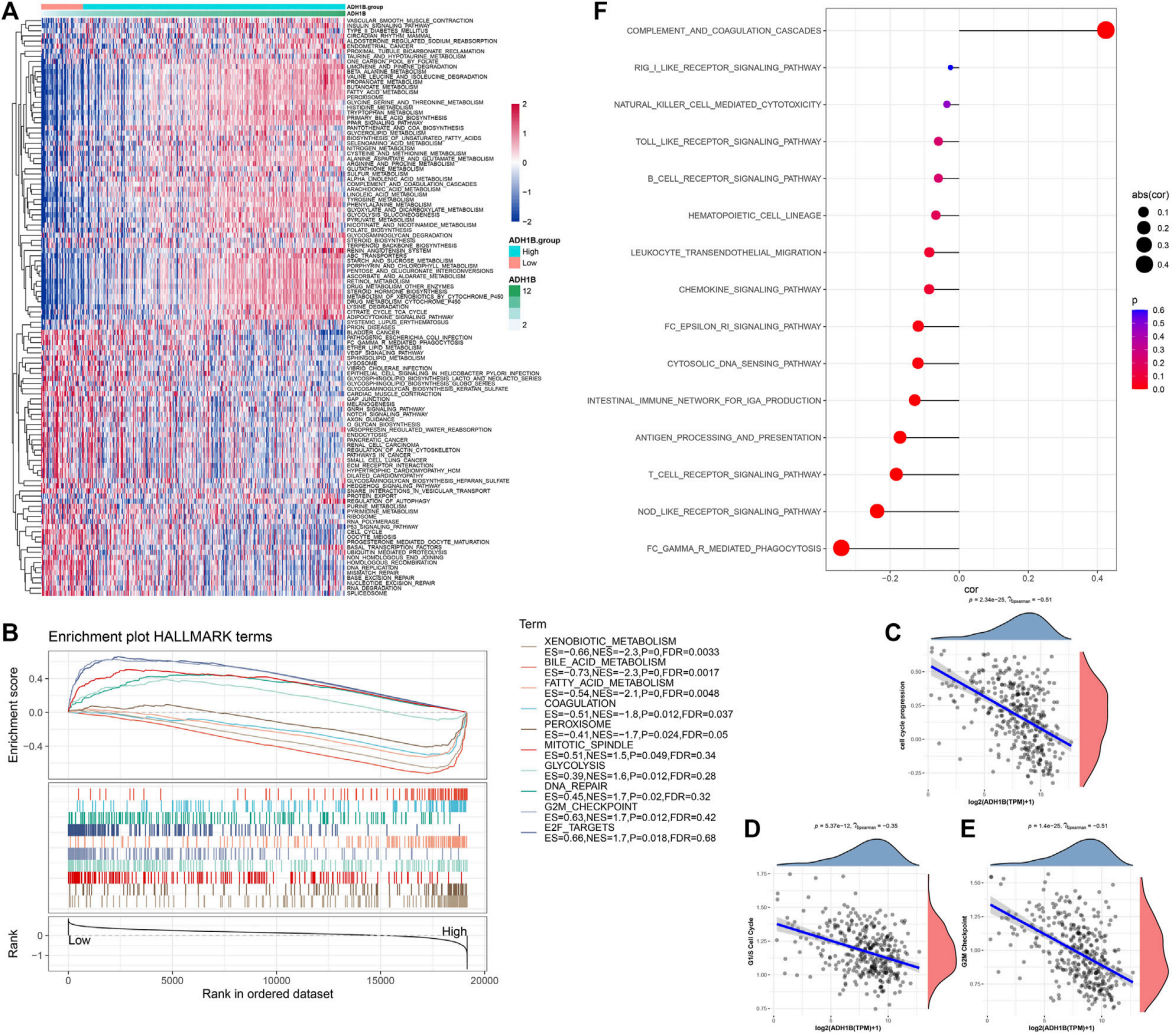
Potential regulatory pathways of ADH1B. **(A)** Differential enriched KEGG pathways between ADH1B-higher-expression group and ADH1B-lower-expression group. **(B)** GSEA showed significant enriched hallmark terms between higher ADH1B and lower ADH1B groups. **(C)** Scatter plots of correlation analysis between the CCP score and ADH1B. **(D)** Scatter plots of correlation analysis between G1/S cell cycle and ADH1B. **(E)** Scatter plots of correlation analysis between G2M Checkpoint and ADH1B. **(F)** Scatter plots of correlation analysis between inflammation pathways and ADH1B.

Subsequently, we assessed the potential regulatory pathways of ADH1B. Figure 5A shows the significant enriched KEGG pathways in the ADH1B-higher-expression group and ADH1B-lower-expression group. We found that VEGF_SIGNALING, P53_SIGNALING_PATHWAY, CELL_CYCLE, and DNA_REPLICATION were significantly activated in the ADH1B-lower-expression group, while FATTY_ACID_METABOLISM and some amino metabolism pathways were enriched in the ADH1B-higher-expression group (Figure 5A). Through GSEA, we also found that HALLMARK_MITOTIC_SPINDLE, HALLMARK_GLYCOLYSIS, HALLMARK_DNA_REPAIR, HALLMARK_G2M_CHECKPOINT, and HALLMARK_E2F_TARGETS were significantly enriched in the ADH1B-lower-expression group, while HALLMARK_XENOBIOTIC_METABOLISM, HALLMARK_BILE_ACID_METABOLISM, HALLMARK_FATTY_ACID_METABOLISM, HALLMARK_COAGULATION, and HALLMARK_PEROXISOME were significantly enriched in the ADH1B-higher-expression group (Figure 5B). The CCP score was negatively correlated with ADH1B (Figure 5C). Thereafter, we found that the G1/S cell cycle and G2M checkpoint were negatively correlated with ADH1B (Figures 5D, E). Meanwhile, most inflammation pathways were negatively correlated with ADH1B (Figure 5F).

### Performance of ADH1B in pan-cancer

We subsequently compared the expression of ADH1B in pan-cancer. The expression level of ADH1B was distinctly downregulated in tumor tissue of each cancer type (Supplementary Figure S1A). We also analyzed the relationship between ADH1B and survival in each cancer type and observed that ADH1B might increase the mortality risk in patients with lung squamous cell carcinoma (LUSC), stomach adenocarcinoma (STAD), and kidney renal papillary cell carcinoma (KIRP) (Supplementary Figure S1B).

### Correlation between ADH1B and its methylation

**FIGURE 6:**
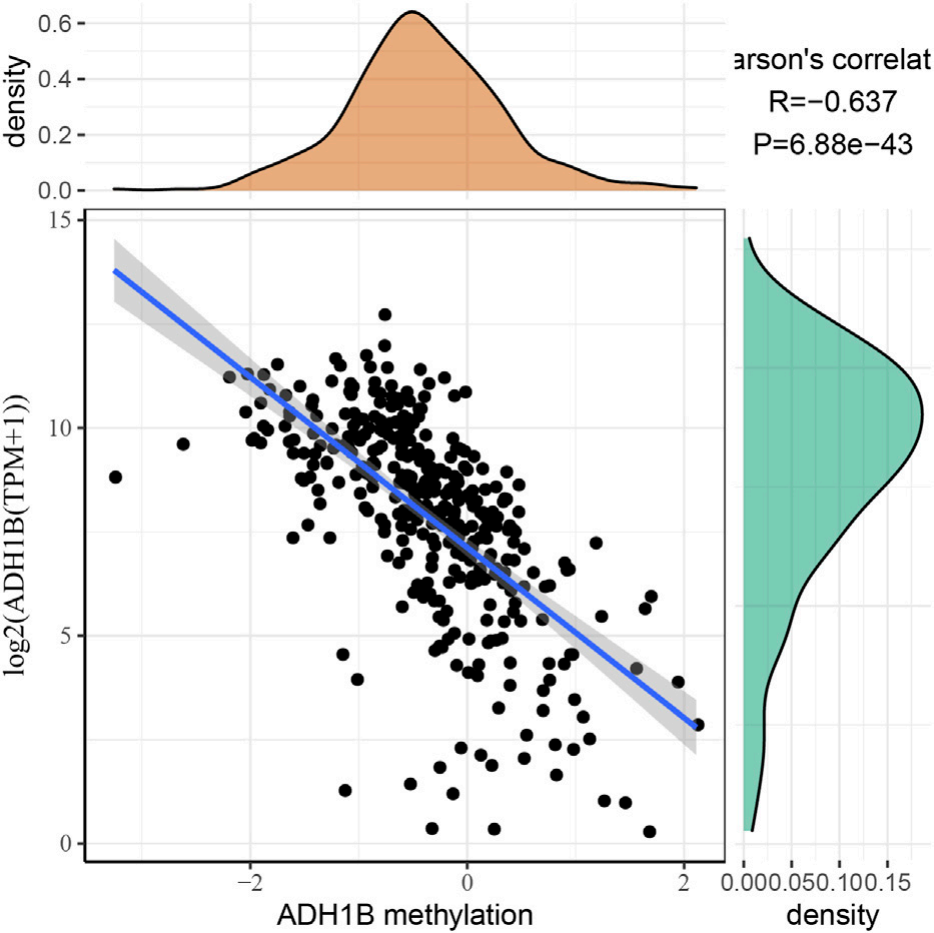
Correlation between ADH1B expression and ADH1B methylation. The expression of the ADH1B gene is negatively correlated with ADH1B methylation.

To further investigate the relationship between ADH1B expression and ADH1B methylation, we obtained methylated data from TCGA and analyzed the correlation using Pearson correlation analysis. As shown in Figure 6, the expression of ADH1B had a significant negative correlation with the methylation of ADH1B (R = −0.637, *p* = 6.88e−43).

### Drug sensitivity analysis of ADH1B

**FIGURE 7:**
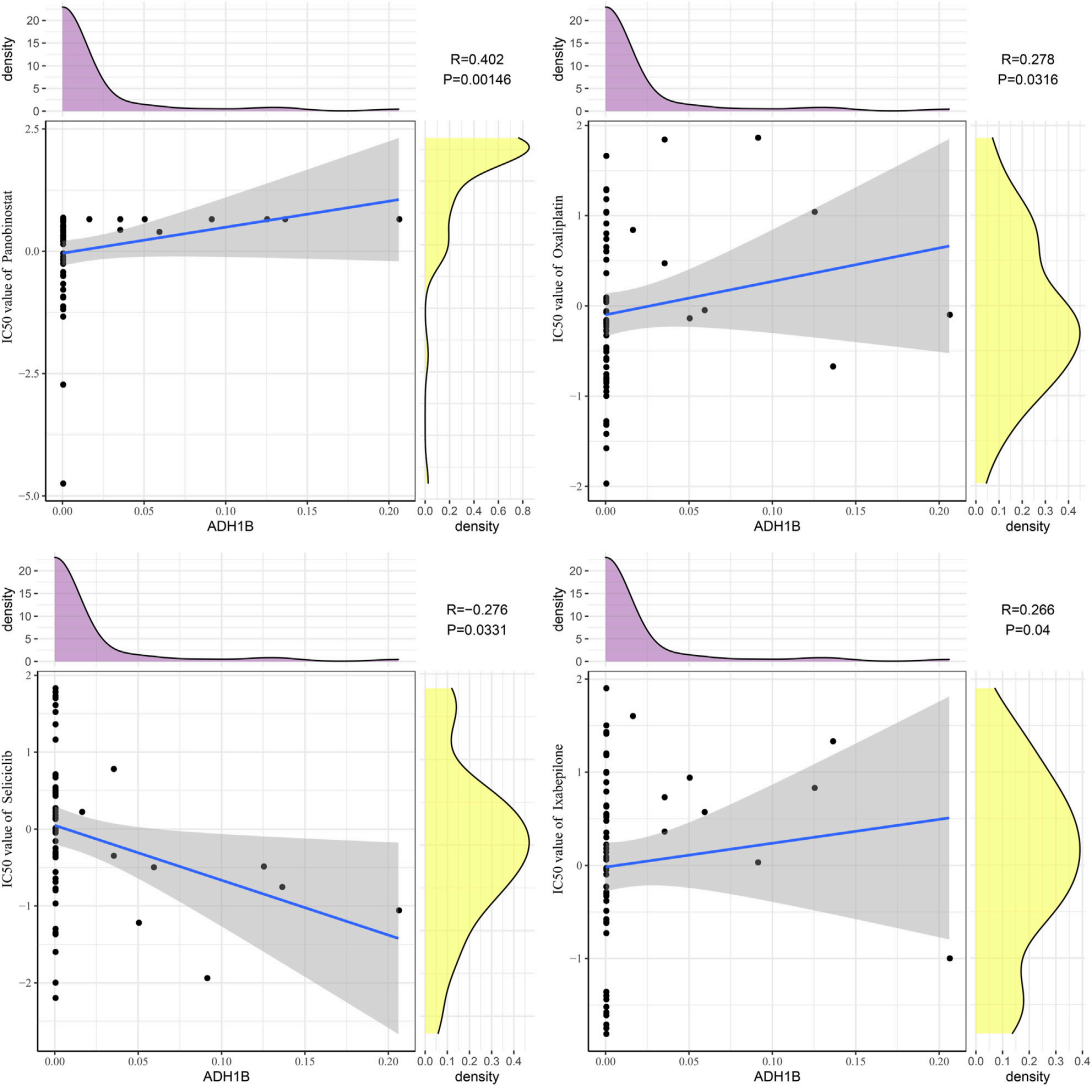
Drug sensitivity analysis of ADH1B. Scatter plots of correlation analysis between ADH1B and drugs (panobinostat, oxaliplatin, ixabepilone, and seliciclib).

Next, we analyzed the relationship between ADH1B and sensitivity to small-molecule drugs. The results revealed that ADH1B was positively correlated with panobinostat (R = 0.402, *p* = 0.00146), oxaliplatin (R = 0.278, *p* = 0.0316), and ixabepilone (R = 0.2666, *p* = 0.04), whereas ADH1B was negatively correlated with seliciclib (R = −0.276, *p* = 0.0331) (Figure 7).

### Decreased expression of ADH1B in hepatocellular carcinoma cell line HepG2

**FIGURE 8:**
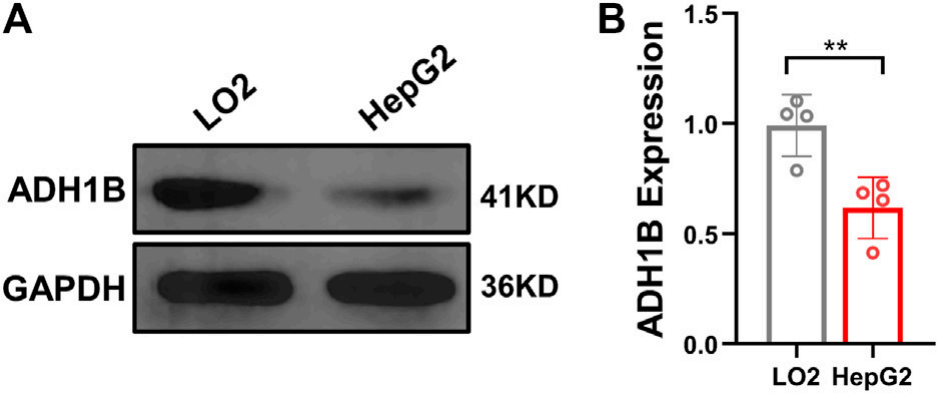
Expression of ADH1B is decreased in the HepG2 cell line. **(A)** Representative Western blot results for ADH1B. **(B)** Quantitative analysis for ADH1B expression.

To validate the expression of ADH1B in LIHC, ADH1B was quantified in normal hepatocytes LO2 and hepatocellular carcinoma cell line HepG2 by using Western blot analysis. As shown in Figure 8, the protein level of ADH1B was significantly decreased in the HepG2 cell line compared with the LO2 cell line (*p < 0.01*).

## Discussion

Using approved drugs to discover innovative biomarkers and potential candidate drugs for specific disorders represents a promising therapeutic approach. In the present study, we screened eight potential candidate genes associated with afatinib between LIHC and normal samples. To identify key genes of afatinib, we analyzed the correlation between immune characteristics and candidate genes. We found ADH1B as a key gene and that patients with lower ADH1B had unfavorable prognosis. Finally, we identified that panobinostat, oxaliplatin, ixabepilone, and seliciclib might be potential drugs in the treatment of LIHC targeting ADH1B.

ASPM is an oncoprotein and activates the EGFR. A previous study has shown that ASPM was highly expressed in glioma cells, and the abnormal expression of ASPM regulated by transcriptional regulation of FoxM1 contributed to the aggressiveness of gliomas (Zeng et al., 2020). In addition, ASPM has been identified as a key gene for HER-2, which is related to the poor prognosis of breast cancer patients (Tjipta et al., 2022). Amplification of CDK4/6 is considered as potential hallmarks for the *de novo* EGFR tyrosine kinase inhibitor (TKI) resistance in sensitizing EGFR mutation NSCLC (Sitthideatphaiboon et al., 2022). Inhibition of CDK4/6 can overcome acquired resistance to third-generation EGFR inhibitor osimertinib in patients with NSCLC (Qin et al., 2020). The upregulated PTMA has been observed in esophageal cancer and LIHC (Zhu et al., 2019; Yang et al., 2021) and served as a potential biomarker associated with progression, early recurrence, and unfavorable prognosis of LIHC (Ha et al., 2015). Aberrant tyrosine catabolic enzyme TAT in patients with LIHC has been found, and a recent study has indicated TAT as a potential gene associated with prognosis of LIHC patients after hepatectomy (Wang et al., 2019). ADH1B belongs to alcohol dehydrogenase class I enzyme and converts ethanol to acetaldehyde via the redox reaction. Downregulated ADH1B has been found in LIHC, and polymorphisms on ADH1B and ALDH2 had distinct indirect functions on the risk of LIHC (Liu et al., 2016). ANXA10 is related to poor prognosis of patients with early gastric cancer, small bowel adenocarcinoma, and lung adenocarcinoma (Ishikawa et al., 2020; Ishikawa et al., 2021; Yumura et al., 2022). As previously reported, the decreased level of ANXA10 was related to vascular invasion, early relapse, and dismal prognosis in synergy with p53 mutation in LIHC (Liu et al., 2002). Additionally, downregulated OGDHL is associated with the advanced tumor stage, unfavorable outcome, and relapse in LIHC through reprogramming glutamine metabolism (Dai et al., 2020). The serum level of PON1 can be considered as a marker to estimate microvascular invasion in patients with LIHC (Ding et al., 2020). Collectively, the eight candidate genes related to afatinib may have great potential in the treatment of LIHC.

The dynamic interplay between tumor cells and the tumor immune microenvironment (TIME) is involved in tumor growth and progression (El-Kenawi et al., 2020). Understanding the TIME is crucial for the mechanism of tumor progression and development of therapeutic strategies (Binnewies et al., 2018). It has been acknowledged that immune cell and inflammatory cell infiltrations are important hallmarks for evaluating the characteristics of the TIME (Ino et al., 2013). Under the conditions of hypoxia, other immune cells, and extracellular matrix, macrophages can reversibly change or alter polarization (Poh and Ernst, 2018). With macrophages, tumor cells invade the circulatory system and escape from cytotoxic lymphocytes and phagocytes through multiple pathways (El-Kenawi et al., 2020). Increased macrophage infiltration is associated with dismal prognosis of cancer patients (Poh and Ernst, 2018). Meanwhile, the interplay between immune cells within a tumor may affect the immunity. Macrophages, monocytes, neutrophils, myeloid-derived suppressor cells (MDSCs), and Tregs exert suppressive effects on cytotoxic lymphocytes (Cassetta and Kitamura, 2018), which promote tumor growth, metastasis, and drug resistance. ADH1B was negatively correlated with several immune cells, such as CD4 T cells, activated dendritic cells, macrophages, mast cells, MDSCs, and Tregs, which contributed to the immunosuppression in LIHC. Moreover, we observed that ADH1B was positively correlated with effector memory CD8 T cells, eosinophils, and type 1 T helper cells. With reduced ADH1B expression, infiltration of CD8 T cells, eosinophils, and type 1 T helper cells decreased, leading to a relevant weak ability of tumor cell killing. These may explain the fact that patients with lower ADH1B had an unfavorable prognosis.

Panobinostat is a histone deacetylase inhibitor that has been first approved for treating refractory multiple myeloma (Eleutherakis-Papaiakovou et al., 2020). Additionally, the combination of panobinostat with Taxol showed synergistic effects on proliferative arrest in head and neck squamous cell carcinoma and NSCLC through inducing senescence (Samaraweera et al., 2017). Panobinostat also had promising activity against other hematologic and solid tumors (Wood et al., 2018; Goldberg et al., 2020). Oxaliplatin is an FDA-approved platinum-based antitumor drug to treat stage III colorectal cancer after tumorectomy and metastatic colorectal cancer. Other indications for oxaliplatin include refractory or relapsed neuroblastoma and non-Hodgkin lymphoma, refractory chronic lymphocytic leukemia, advanced biliary adenocarcinoma, ovarian cancer, and pancreatic cancer (Devanabanda and Kasi, 2022). Ixabepilone is a semi-synthetic analog of epothilone B and serves as a microtubule inhibitor that has been first approved for treating metastatic breast cancer (Ibrahim, 2021). Ixabepilone also shows potential activities against meningioma and platinum/taxane-resistant/refractory ovarian cancer (Roque et al., 2021; Jungwirth et al., 2023). Seliciclib, an oral inhibitor of cyclin-dependent kinases, can be used to treat Cushing disease and cystic fibrosis (Liu et al., 2022; Meijer et al., 2022). At present, seliciclib has been developed as an anticancer medicine for treating NSCLC, nasopharyngeal carcinoma, prostate cancer, metastatic breast cancer, and osteosarcoma (Aldoss et al., 2009; Keenan et al., 2019; Fu et al., 2020; Alsfouk et al., 2021). The current study found that ADH1B was positively correlated with panobinostat, oxaliplatin, and ixabepilone, whereas ADH1B was negatively correlated with seliciclib. Our findings suggested that small-molecule drugs panobinostat, oxaliplatin, ixabepilone, and seliciclib could be potential treatment medicines targeting ADH1B in LIHC.

There are still some limitations in this study. Although we have identified ADH1B as the key gene of afatinib, the underlying mechanism of ADH1B in LIHC remains unclarified. Thus, further experimental studies should be carried out to reveal the mechanism of its action. In addition, we only screened the key gene related to afatinib, and further study on other potential drugs and their related genes to develop potential drugs for the treatment of LIHC should be implemented. Moreover, we used CellMiner to screen the potential anticancer drugs for LIHC in this study, other approaches such as network pharmacology and protein–protein interaction network should be used for drug repurposing for LIHC.

## Conclusion

We screened eight potential candidate genes associated with afatinib, ASPM, CDK4, PTMA, TAT, ADH1B, ANXA10, OGDHL, and PON1. Then, we identified ADH1B as a key gene based on immune abnormalities, which could predict the prognosis of LIHC patients and negatively correlated with cell cycle progression and inflammation pathways. We further found that small-molecule drugs panobinostat, oxaliplatin, ixabepilone, and seliciclib were significantly associated with ADH1B, which might be potential drugs targeting ADH1B for LIHC management.

## Data Availability

The original contributions presented in the study are included in the article/Supplementary Material; further inquiries can be directed to the corresponding author.

## Abbreviations

LIHC, liver hepatocellular carcinoma; EGFR, epidermal growth factor receptor; TKI, tyrosine kinase inhibitor; ERK, extracellular signal-regulated kinase; VEGF, vascular endothelial growth factor; MMP, matrix metalloproteinase; TCGA, The Cancer Genome Atlas; GEO, Gene Expression Omnibus; HCCDB, Hepatocellular Carcinoma Database; GDSC, Genomics of Drug Sensitivity in Cancer; HBV, hepatitis B virus; HCV, hepatitis C virus; KEGG, Kyoto Encyclopedia of Genes and Genomes; ssGSEA, single-sample gene set enrichment analysis; IC50, half-maximal inhibitory concentration; FDR, false discovery rate; LUSC, lung squamous cell carcinoma; STAD, stomach adenocarcinoma; KIRP, kidney renal papillary cell carcinoma; MDSCs, myeloid-derived suppressor cells; TIME, tumor immune microenvironment.
