# Supplementary material for: Identification of afatinib-associated ADH1B and potential small-molecule drugs targeting ADH1B for hepatocellular carcinoma
Source: Front Pharmacol. 2023 May 9;14:1166454. doi: 10.3389/fphar.2023.1166454 (PMC10203513; doi:10.3389/fphar.2023.1166454)
Supplement: Supplementary file 1 [file DataSheet1.docx]

**Supplementary Fig 1** Performance of ADH1B in pan-cancer. A, ADH1B was distinctly downregulated in tumor tissue of each cancer type. B, Forest plot showed the relationship between ADH1B and survival in each cancer type. ^****^*P* <0.0001.
