# Supplementary figures and images for: Identification of afatinib-associated ADH1B and potential small-molecule drugs targeting ADH1B for hepatocellular carcinoma
Source: Front Pharmacol. 2023 May 9;14:1166454. doi: 10.3389/fphar.2023.1166454 (PMC10203513; doi:10.3389/fphar.2023.1166454)

A

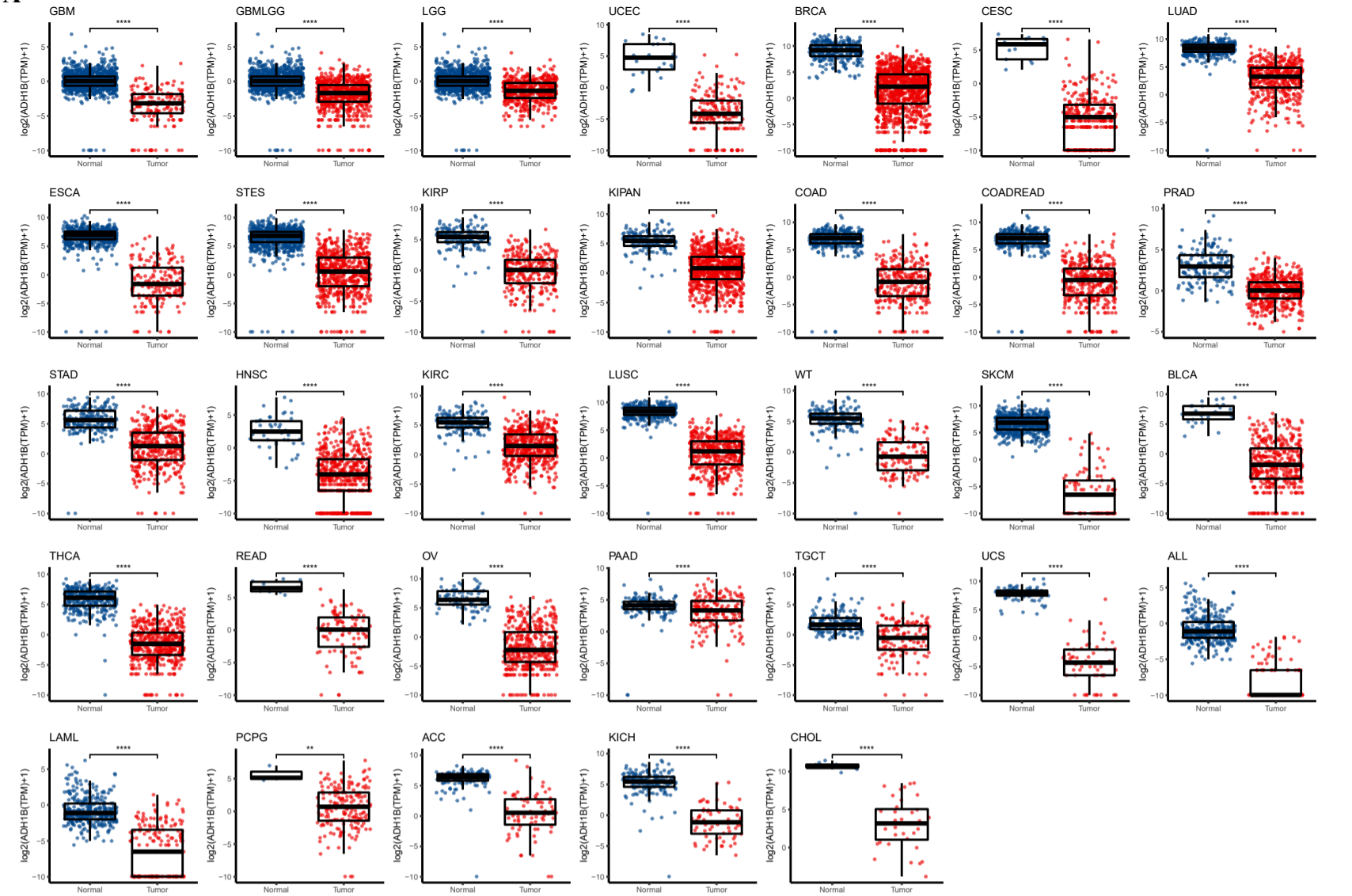

B

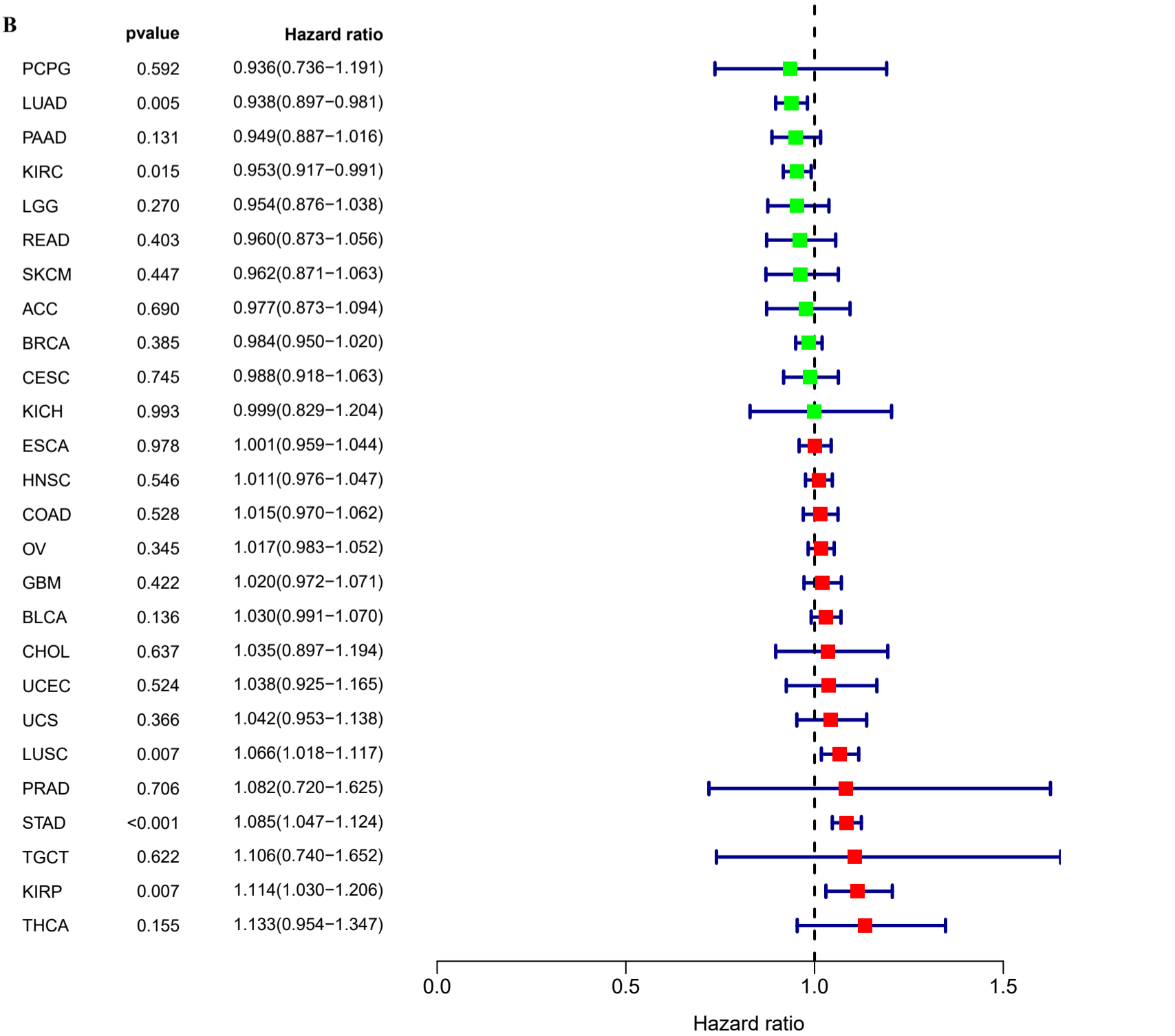

Supplement: Supplementary file 2 [file Image1.pdf]
